# An Ovarian Sertoli–Leydig Cell Tumor with Elevated Alpha-Fetoprotein in an Adolescent: A Rare Case Report and Literature Review

**DOI:** 10.3390/medicina60091477

**Published:** 2024-09-10

**Authors:** Gabija Žilinskienė, Diana Bužinskienė, Evelina Šidlovska, Vilius Rudaitis

**Affiliations:** 1 Faculty of Medicine, Vilnius University, LT-03101 Vilnius, Lithuania; diana.buzinskiene@mf.vu.lt (D.B.); vilius.rudaitis@mf.vu.lt (V.R.); 2 Clinic of Obstetrics and Gynaecology, Institute of Clinical Medicine, Faculty of Medicine, Vilnius University, LT-03101 Vilnius, Lithuania; 3 National Center of Pathology, Affiliate of Vilnius University Hospital Santaros Klinikos, LT-08406 Vilnius, Lithuania; evelina.sidlovska@vpc.lt

**Keywords:** alpha-fetoprotein, amenorrhea, ovarian tumor, Sertoli–Leydig cell tumor

## Abstract

An ovarian Sertoli–Leydig cell tumor is a rare type of sex cord–stromal tumor of the ovary. Typically, it presents as abdominal pain or androgenic manifestations in women in the second to third decade of life. While cases of ovarian Sertoli–Leydig cell tumor associated with increased levels of alpha-fetoprotein are rare, they are reported to be the most common alpha-fetoprotein-producing ovarian non-germ cell tumor. We report the case of a 16-year-old patient, who presented with complaints of amenorrhea that had lasted for one year. Transabdominal ultrasound revealed the presence of a tumor in the right ovary, measuring 9.3 × 5.8 cm in size. The laboratory investigation showed an increased level of alpha-fetoprotein. The patient underwent laparoscopic right salpingo-oophorectomy. Histopathological examination confirmed the presence of a moderately differentiated (G2) Sertoli–Leydig cell tumor in the right ovary. For reproductive-age patients with disease confined to the ovary, fertility-sparing surgery is recommended. According to the current recommendations, the administration of adjuvant chemotherapy is indicated in cases of the presence of heterologous elements, poorly differentiated tumors, or FIGO stages IB–IV. As there were no high-risk factors and no residual disease in this case, there were no indications for further treatment with adjuvant chemotherapy. A recent follow-up visit showed that the patient is in complete remission. This report presents a detailed description of the findings, differential diagnosis, clinical course, chosen treatment, and prognosis. Also, a comprehensive literature review of ovarian Sertoli–Leydig cell tumors, focusing on their clinical presentation, laboratory findings, macroscopic and histopathological features, genetics, clinical management, prognostic factors and follow-up, is provided.

## 1. Introduction

Ovarian Sertoli–Leydig cell tumors (SLCTs), also known as androblastoma or arrhenoblastoma, belong to the group of sex cord–stromal tumors, which demonstrate a testicular pattern of differentiation [1]. SLCTs are rare, accounting for less than 0.5% of all ovarian neoplasms [2].

Due to their rarity, the information about these tumors is mostly acquired from small case series and retrospective reviews [3]. SLCTs occur in reproductive-age women, usually 30 years of age or younger [2,4,5,6]. The typical clinical presentation is abdominal pain or hormone-related symptoms [1,3,4,6,7,8,9]. It is reported that approximately one-third of SLCTs are associated with androgen-excess manifestations, such as oligomenorrhea, amenorrhea, hirsutism, clitoromegaly, and breast atrophy [9]. Estrogenic hormonal manifestations, such as abnormal uterine bleeding or postmenopausal bleeding, are rare [8]. In most cases, SLCTs are confined to a unilateral ovary and are voluminous [1,6,9]. Based on the largest clinicopathological series to date, including 207 ovarian SLCTs, 1.5% of tumors were bilateral and only 2.5% were beyond stage I [7]. According to the World Health Organization (WHO)’s suggested classification, SLCTs can be organized into four histological types: well differentiated, moderately differentiated, poorly differentiated, and retiform; in a fifth of neoplasms of the latter three types, heterologous elements can be found [5,8,9,10]. Moreover, Sertoli–Leydig cell tumors are known to have an association with the *DICER1* gene and can be part of the clinical spectrum of *DICER1* syndrome [3,10,11]. The reported frequency of *DICER1* mutations in SLCTs ranges from 15% to 97% [12]. It is noticed that *DICER1* mutation is only common in moderately and poorly differentiated SLCTs, whereas well-differentiated SLCTs appear to be *DICER1*-independent [11,12,13,14]. In addition, germline and somatic *DICER1* mutations tend to be more common in patients younger than 18 years old [5,10,11,12,14,15]. While cases of ovarian SLCT associated with increased levels of alpha-fetoprotein (AFP) are rare, they are reported to be the most common alpha-fetoprotein-producing ovarian non-germ cell tumor [16]. The FIGO (International Federation of Gynaecology and Obstetrics) stage and degree of differentiation are the most important prognostic factors [5,7,10,17,18]. It is known that the presence of a retiform component or mesenchymal heterologous elements may be associated with unfavorable prognosis [5,7,10].

This paper describes a rare case of unilateral Sertoli–Leydig cell ovarian tumor with AFP elevation that caused amenorrhea in an adolescent patient and presents a comprehensive literature review of ovarian Sertoli–Leydig cell tumors, focusing on their clinical presentation, laboratory findings, macroscopic and histopathological features, genetics, clinical management, prognostic factors and follow-up, which adds practical relevance and might aid clinicians in decision-making.

## 2. Case Report

A 16-year-old adolescent (parity 0, abortions 0, miscarriages 0) presented to a gynecologist with a complaint of amenorrhea that had lasted for one year.

It is known that menarche occurred at the age of 12 years. The patient did not have isosexual pseudoprecocity or precocious puberty. The patient reported that her menstrual cycle was more or less regular before, and there was not such a long period of amenorrhea. She was otherwise healthy, with no other complaints or diseases. The patient had no family history of breast, ovarian, or uterine cancer.

**Figure 1:**
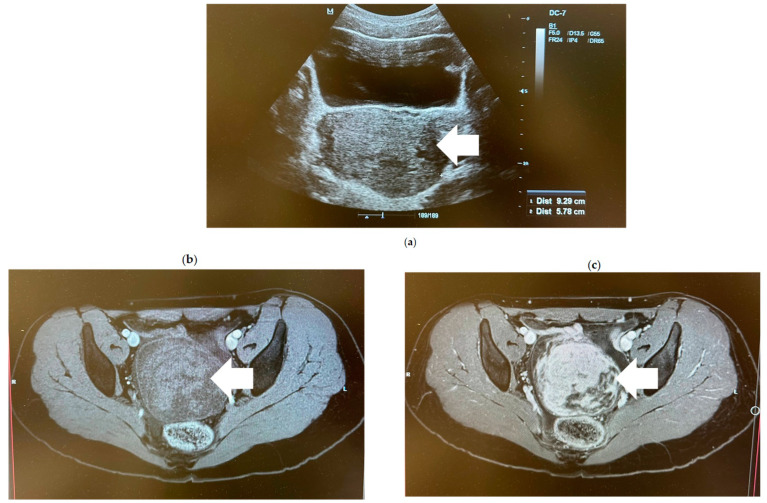
Radiologic evaluation. (**a**) Transabdominal ultrasound image showing a solid mass (arrow) in the right ovary. (**b**,**c**) Axial pelvic MRI images showing the right ovarian heterogenic structure of a solid mass (arrow) with a capsule that enhanced intensely after the administration of contrast medium.

At presentation, her height was 165 cm (50th percentile), weight 58 kg (50th percentile), and BMI 21.3 (60th percentile). On physical examination, her abdomen was soft and not painful, but in the region of the right ovary, a 10 cm sized painless pelvic mass was palpated. The patient’s pubic hair and breast tissue showed puberty was fully completed and were equivalent to Tanner stage 5. The external genitalia were without any clearly visible lesions, with clear physiological discharge, and the clitoris, labia minora and majora were not enlarged. Other physical assessments were not clinically significant. No signs of virilization were clinically detected. The patient’s Ferriman Gallwey score was 4, which consisted of 1 point each for upper lip, chest, lower abdomen and upper arms. Transabdominal ultrasound revealed the presence of well-defined tumor in the right ovary, measuring 9.3 × 5.8 cm in size (Figure 1a). A pelvic magnetic resonance imaging (MRI) scan confirmed the presence of the heterogenic structure of a solid mass in the right ovary (Figure 1b,c), and the Ovarian-Adnexal Reporting and Data System Magnetic Resonance Imaging (O-RADS MRI) score for that mass was 5—high risk. Moreover, free fluid was observed in the pelvis.

The laboratory investigation showed an increased level of alpha-fetoprotein (AFP) (800 IU/mL) (normal range: 0.5–5.5 IU/mL), whereas the patient’s serum levels of cancer antigen 125 (CA-125), human chorionic gonadotropin (*β-hCG*) and testosterone were within normal values. The presence of a unilateral ovarian mass with the features of malignancy in an adolescent girl, whose only abnormal laboratory finding was the presence of raised serum levels of AFP, led to the suspicion of an ovarian germ cell tumor, such as a yolk sac tumor.

**Figure 2:**
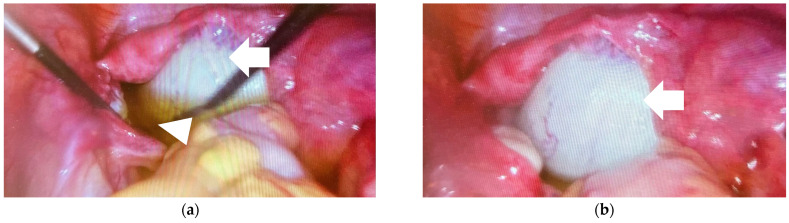
Laparoscopic evaluation. (**a**,**b**) Right ovarian tumor (arrow) and yellowish free fluid in the pelvis (arrowhead).

Because the tumor had MRI features suggestive of malignancy and was evaluated as high risk, the patient underwent laparoscopic right salpingo-oophorectomy (Figure 2a,b). The contents of the tumor did not enter the peritoneal cavity. During the operation, yellowish pelvic free fluid was aspirated. There were no other visible pathologic changes in the abdominal cavity.

**Figure 3:**
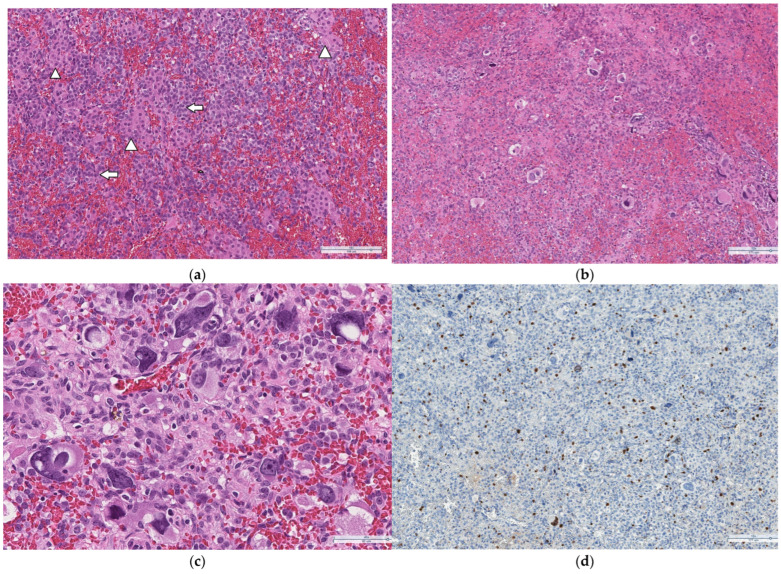

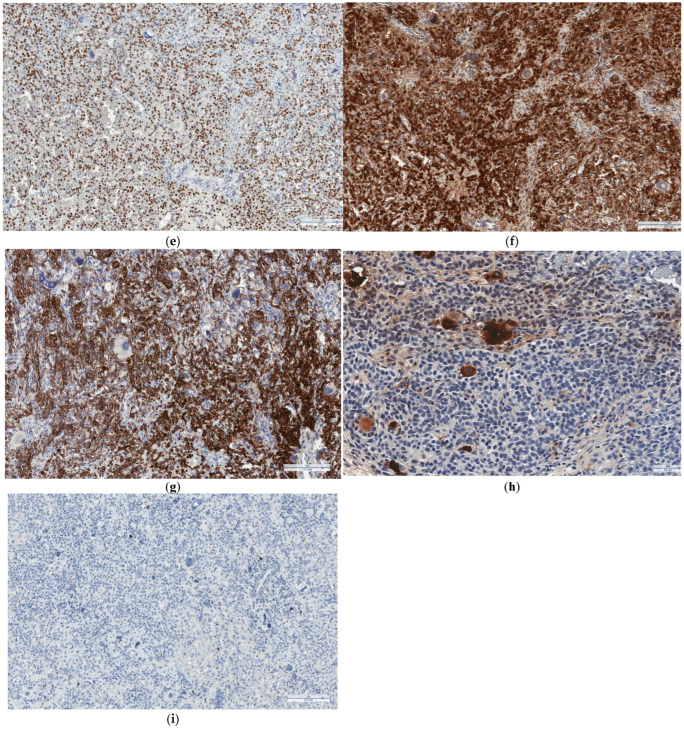
Microscopic evaluation. (**a**) Sertoli–Leydig cell tumor composed of 2 cell types: Sertoli (arrow) and Leydig (arrowhead). (**b**) Areas of degenerative atypia: bizarre cells with large, pleomorphic, irregularly contoured, hyperchromic nuclei with abundant intranuclear inclusions and perinuclear cytoplasmic condensation. (**c**) Pleomorphic cells at higher magnification. (**d**) Figure demonstrating Ki-67 staining in the tumor: nuclear positivity in 7% of Sertoli–Leydig cells and only single positive nuclei in pleomorphic cells. (**e**) SF1 nuclear staining with moderate intensity in Sertoli–Leydig cells and is negative in pleomorphic cells. (**f**) Immunohistochemical analysis of the Sertoli–Leydig cell tumor (positive stain of calretinin in Sertoli cell and Leydig cell). (**g**) Immunohistochemical analysis of the Sertoli–Leydig cell tumor (positive stain of α-inhibin in Sertoli cell and Leydig cell). (**h**) AFP showing staining in sporadic pleomorphic cells. (**i**) SALL4 staining showing a completely negative reaction in all the tumor cells.

No atypical cells were found in the cytological examination of the aspirated fluid. Histopathological examination confirmed the presence of a moderately differentiated (G2) Sertoli–Leydig cell tumor, measuring 12 × 10 × 8 cm, in the right ovary, pT1a (tumor limited to one ovary) and LVI0. Microscopically, the right ovarian tumor was composed of solid areas and small cystic structures. The solid areas forming trabecular structures, single tubules and small sheets were composed of Sertoli cells with scanty eosinophilic cytoplasm and hyperchromatic, oval nuclei. Clusters and single cells with abundant eosinophilic cytoplasm with lipofuscin pigment and round central nuclei consistent with Leydig cells were identified between the trabeculae and sheets of Sertoli cells (Figure 3a). The tumor contained small areas with signs of degenerative atypia: giant cells with large, pleomorphic, irregularly contoured, hyperchromic nuclei with abundant intranuclear inclusions and perinuclear cytoplasmic condensation (Figure 3b,c). There were 4–5 mitosis observed per 10 high-power fields (HPFs). The Ki67 value was 7% (Figure 3d). Immunohistochemical staining showed positivity for steroidogenic factor 1 (SF1), calretinin (Figure 3f), inhibin (Figure 3g), and in sporadic cells, for AFP (Figure 3h), while staining for SALL4 (Figure 3i), which is known to be a malignant germ cell tumor marker, was negative. Histological evidence of hepatoid differentiation was absent. Additional HEPA and HCG stainings were completely negative.

**Figure 4:**
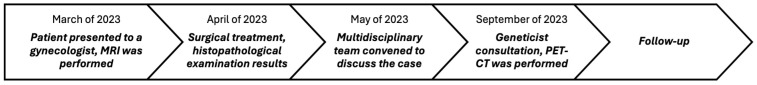
Timeline with relevant data from the episode of care.

The patient’s postoperative recovery was uneventful. Three weeks after the operation, menstruation reappeared. The repeated serum AFP level was within the normal range. A multidisciplinary team (MDT), which consisted of a gynecologist, radiation oncologist, medical oncologist, and radiologist, convened to consider the best route of care for this rare case. A positron emission tomography–computed tomography (PET-CT) scan was performed, which did not detect any nodal or distant disease. A geneticist consultation was planned to rule out or confirm a *DICER1* mutation, and no mutation was found. As there were no high-risk factors and no residual disease in this case, there were no indications for further treatment with adjuvant chemotherapy. However, close follow-up is needed within the first five years after diagnosis. The follow-up visits, which are planned for every six months, include a detailed history, physical examination, serum testosterone, AFP levels and ultrasound examination of the abdomen and pelvis. In addition, once a year, pelvic MRI is scheduled. A recent follow-up visit showed that the patient is in complete remission (Figure 4).

## 3. Discussion and Review of the Literature

### 3.1. Clinical Features of Ovarian Sertoli–Leydig Cell Tumor

Based on the WHO’s classification, ovarian Sertoli–Leydig cell tumors are a subgroup of ovarian sex–chord stromal tumors [19]. SLCTs are rare, as their specific incidence is reported to be certainly lower than the 2.1/1,000,000 women yearly adjusted incidence rate reported for the whole group of ovarian sex cord–stromal tumors [4].

They are most common in reproductive-age patients in the second to third decade of life [2,4,5,6]. However, some studies reported that SLCTs were also seen in postmenopausal women [2,4,9]. The leading clinical symptoms are abdominal pain and androgen-excess manifestations, such as oligomenorrhea, amenorrhea, and symptoms of virilization [1,3,4,6,7,8,9]. Diagnosis of SLCT is particularly challenging since it can occur in patients older than the typical age and more than half of all cases lack androgenic manifestations [2,4,9]. In our presented case, the only complaint was amenorrhea, and no external androgen-excess features were detected.

### 3.2. Laboratory Findings, Tumor Macroscopic and Histological Features, and Differential Diagnosis

Approximately half of these tumors are associated with evidence of androgen excess, whereas estrogenic hormonal manifestations are rare [2].

AFP is a major plasma glycoprotein and an important tumor marker for related malignancies, such as hepatocellular carcinoma and germ cell tumors [9,20]. While cases of ovarian Sertoli–Leydig cell tumors associated with raised levels of alpha-fetoprotein are rare, they are reported to be the most common alpha-fetoprotein-producing ovarian non-germ cell tumor [16].

The average SLCT diameter is 13.5 cm, but it might be much more voluminous in poorly differentiated histological variants [1]. The components of SLCTs can be purely solid, purely cystic, or mixed [1]. A mixed (solid and cystic) structure, as in our presented case, is most common in ovarian SLCTs and is found in almost two-thirds of cases [1]. Microscopically, SLCTs are composed of variable combinations of Sertoli and Leydig cells, surrounded by other possible components [9]. SLCTs can be organized into four histological types: well differentiated, moderately differentiated, poorly differentiated, and retiform; in the latter three types, heterologous elements can be found [10]. Heterologous elements are arranged into two types: endodermal elements, of which the most common is gastrointestinal epithelium, and mesenchymal elements, e.g., cartilage or skeletal muscle [9]. Poor differentiation and the presence of a retiform subtype or heterologous elements are histologic indicators of a poor prognosis [3]. Tumors with heterologous mesenchymal elements are associated with a poor outcome, whereas the occurrence of gastrointestinal epithelium elements does not alter the prognosis [1]. In our reported case, no heterologous elements were detected. Focal AFP staining is common in SLCT, which complicates differentiating it from germ cell tumors [5,21] (Table 1).

In our described clinical case, the laboratory findings showed a significant increase in AFP, while immunohistochemical staining showed positivity for AFP in sporadic cells, but histological evidence of hepatoid differentiation was absent and the immunohistochemical marker for a malignant germ cell tumor was negative. The origin of AFP-producing cells in SLCTs is still under consideration. In some cases, AFP production is linked to the presence of a heterologous element, such as intestinal-type mucinous epithelium or hepatocytes [22]. In cases with no heterologous element, like our current case, the most frequent hypothesis is that AFP-producing cells are Leydig-like cells [6,7,8,16].

### 3.3. Genetics

Both germline and somatic *DICER1* gene mutations can be found in ovarian SLCTs [11]. A germline mutation of the *DICER1* gene causes the rare eponymous autosomal dominant syndrome predisposing the development of multiple tumor types, e.g., pleuropulmonary blastoma, cystic nephroma, and ovarian SLCT [11]. It is noticed that *DICER1* mutations are more commonly found in younger patients with SLCT [10]. According to recent studies on the *DICER 1* mutation status in ovarian Sertoli–Leydig cell tumors by Kato et al., Karnezis et al., and Yang et al., in a group of patients aged 18 year-old and younger, 94% (17/18) of cases were found to harbor a *DICER1* mutation [12,15,23]. *DICER1* mutation appears to be restricted to moderately or poorly differentiated tumors [11]. In our case, the patient was otherwise healthy and had no history of other tumors, such as kidney, thyroid, brain, or lung rumors. However, according to previously mentioned information about the common patient age and grade of tumor differentiation, in this case, *DICER1* mutation was highly suspected. It is assumed that *DICER1* mutations are involved in the dysregulation of sex hormone synthesis and are closely associated with androgenic manifestations in cases of ovarian SLCTs [15]. It is thought that *DICER1* mutation is associated with reduced expression of an enzyme that is essential for the biosynthesis of estrogen through its role in converting testosterone to estradiol [11]. Considering the variation in the frequency of *DICER1* mutations reported in SLCTs, it has been suggested that the *DICER1* mutation status may have value as a prognostic rather than a diagnostic marker [14]. Moreover, the literature data about its prognostic value are controversial. On the one hand, it is reported that patients with *DICER1* germline mutations may be more likely to exhibit clinical relapse and have poorer prognosis [10]. On the other hand, there are suggestions that the overall and disease-free survival are better among patients with germline than somatic mutations of *DICER1* [11].

### 3.4. Treatment

When planning treatment for patients with SLCT, the most important factors are the patient’s age and cancer stage [10]. Fertility-sparing surgery is recommended as a safe and effective treatment for young patients with FIGO stage IA [1,8,10,17,18,19]. For postmenopausal women and in cases of FIGO IB or more advanced disease, the ESMO (European Society for Medical Oncology) recommends hysterectomy and bilateral salpingo-oophorectomy [19]. It is very important to avoid tumor spillage since it has been associated with an increased risk of recurrence [18]. Recent publications (provided following) on the incidence of lymph node metastases in sex cord–stromal tumors (SCTSs) confirm that lymph node metastasis are rare in these tumors and support the position that lymphadenectomy is not needed and may be omitted when staging patients with ovarian SCTs [19,24,25]. As in our presented case there were no suspicious lymph nodes observed on the pelvic MRI that was performed before surgery and no visible pathologic changes were detected in the abdominal cavity during the surgical treatment, lympho-nodal dissection was not performed. For patients with early-stage disease, the indications for the administration of adjuvant chemotherapy are not clearly determined [18]. According to the current data, patients with high-risk factors (poor differentiation, stage greater than IA, tumor rupture or intraoperative spillage, presence of heterologous elements) should receive adjuvant chemotherapy [7,8,10,17,18]. In addition, there are some recommendations that adjuvant chemotherapy should be proposed when a retiform pattern is present [10,17]. The recently updated ESMO guidelines on the management of non-epithelial ovarian tumors recommend the administration of adjuvant chemotherapy for patients with SLCTs in the presence of heterologous elements, poorly differentiated tumors or FIGO stages IB–IV [19]. The necessity of adjuvant chemotherapy in our presented case of a stage 1A, moderately differentiated SLCT without a retiform pattern or heterologous elements is still unclear [8]. Therefore, in every single case, it should be weighted whether the risk of chemotherapy agents’ potential side effects is higher than the possible benefits. As there were no high-risk factors and no residual disease in our case, there were no obvious benefits observed for further treatment with adjuvant chemotherapy.

### 3.5. Prognostic Factors and Follow-Up

It is reported that the FIGO stage and degree of differentiation are the most important prognostic factors [5,17]. The presence of a retiform pattern and mesenchymal heterologous elements have been associated with an unfavorable prognosis [5,7,17]. In the largest cohort of patients diagnosed with advanced SLCTs, women with extra-ovarian disease had over two times worse 5-year cancer-specific survival (CSS) rates compared to those with SLCTs confided to the ovary (40.8% and 87.6%, respectively) [18]. According to the reviewed literature, for our patient with an early-stage SLCT and the absence of high-risk factors, the prognosis is favorable. The reported recurrence rates vary from 0 to 33.3% [18]. Patients with SLCTs tend to relapse early [18]. Cases of recurrence are reported to have a low survival rate [7,18]. Therefore, close follow-up in cases of ovarian SLCTs is required [7,18]. During follow-up visits, a detailed history, physical examination, laboratory tests, such as serum testosterone, AFP levels, and ultrasound examination of the abdomen and pelvis need to be performed.

## 4. Conclusions

An ovarian Sertoli–Leydig cell tumor is a rare type of sex cord–stromal tumor of the ovary. The rarity of Sertoli–Leydig cell tumors leads to a low index of suspicion; therefore, in-depth knowledge of the clinicopathological and immunological characteristics of such tumors is crucial for their diagnosis and proper management of the treatment and follow-up. An SLCT may be suspected clinically in a young patient presenting with a combination of androgen excess symptoms and voluminous ovarian 297 mass on imaging studies. We presented a rare clinical case of an early-stage moderately differentiated unilateral DICER1 wild-type Sertoli–Leydig cell ovarian tumor with AFP elevation that caused amenorrhea in an adolescent patient. In our case, the main differential diagnosis was a yolk sac ovarian tumor. The patient underwent successful fertility-sparing surgical treatment, and there were no indications for further treatment with adjuvant therapy. Considering the circumstances, namely that our patient presented with early-stage disease and the absence of high-risk factors, her prognosis is favorable.

## Figures and Tables

**Table 1:** Differential diagnosis.

	Yolk Sac Ovarian Tumor	Sertoli–Leydig Ovarian Tumor
Clinical presentation	Acute abdomen, ovarian torsion, precocious puberty, abdominal pain or distention, palpable mass	Pelvic pain, pelvic mass, androgen-excess manifestations, estrogen-excess manifestations (rare)
Hormonal activity	-	Androgenic, estrogenic (rare)
Behavior	Malignant	Correlates with tumor grade and histologic subtype
Serum markers	Alpha fetoprotein (AFP)	Elevated testosterone (+/−), Alpha fetoprotein (AFP) (rare)
Immunohistochemical marker	SALL4 (+), AFP (+),	Inhibin (+), calretinin (+), SF1 (+), AFP (+/−), SALL4 (−)

## Data Availability

The original contributions presented in the study are included in the article, further inquiries can be directed to the corresponding author.
